# Explaining racial-ethnic differences in hypertension and diabetes control among veterans before and after patient-centered medical home implementation

**DOI:** 10.1371/journal.pone.0240306

**Published:** 2020-10-12

**Authors:** Lucinda B. Leung, W. Neil Steers, Katherine J. Hoggatt, Donna L. Washington

**Affiliations:** 1 VA HSR&D Center for the Study of Healthcare Innovation, Implementation, & Policy (Health Equity-QUERI National Partnered Evaluation Center), VA Greater Los Angeles Healthcare System, Los Angeles, California, United States of America; 2 Division of General Internal Medicine and Health Services Research, Department of Medicine, UCLA David Geffen School of Medicine, Los Angeles, California, United States of America; 3 San Francisco VA Health Care System, San Francisco, California, United States of America; 4 Department of Medicine, University of California, San Francisco, San Francisco, CA, United States of America; Montana State University, UNITED STATES

## Abstract

Patient-centered medical homes (PCMH) are primary care delivery models that improve care access and population-level health outcomes, yet they have not been observed to narrow racial-ethnic disparities in the Veteran Health Administration (VHA) or other health systems. We aimed to identify and compare underlying drivers of persistent hypertension and diabetes control differences between non-Hispanic Black (Black) and Hispanic versus non-Hispanic White (White) patients before and after PCMH implementation in the VHA. Among Black and Hispanic versus White VHA primary care patients in 2009 (n_hypertension_ = 26,906; n_diabetes_ = 21,141) and 2014 (n_hypertension_ = 83,809; n_diabetes_ = 38,887), we retrospectively examined hypertension control (blood pressure<140/90) and diabetes control (hemoglobin A1c <9) obtained through random chart abstraction of patient health records nationally via VHA’s quality monitoring program. We fit linear probability regression models, adjusting for age, gender, comorbidity, and socioeconomic status (SES). Blinder-Oaxaca and Smith-Welch decomposition methods were used to parse out explained and unexplained contributors to health disparity between racial-ethnic groups pre- and post-PCMH implementation. Compared to White patients, hypertension and diabetes control remained significantly lower for Black (-6.2%[0.4%] and -3.1%[0.6%], respectively; p’s<0.001) and Hispanic (-1.4%[0.8%] and -4.0%[1.0%], respectively; p’s<0.001) patients following VHA PCMH implementation. Most racial-ethnic differences (55.7–92.3%; all p<0.05) were not attributed to age, gender, comorbidity, and SES. The contribution of explained versus unexplained factors did not significantly change over time. While many explanations for persistent racial-ethnic disparities in disease control among veterans exist, our study did not find that it was due to an influx of “sick” or “socioeconomically vulnerable” patients into the VHA following PCMH implementation. Instead, unexplained differences may be due to differential healthcare and community experiences (e.g., discrimination). Understanding underlying pathways leading to health disparities will better inform policy and clinical interventions to improve PCMH care delivery to racial-ethnic minority patients in health systems.

## Introduction

Multilevel factors (i.e., patient, clinical, administrative, health professional, organizational, legal, and health system regulations) contribute to the persistence of racial-ethnic disparities in important health outcomes among United States (US) primary care patients [1]. For Black non-Hispanic (Black) and Hispanic groups compared to White non-Hispanic (White) patients, chronic disease prevalence has been found to be higher, but quality of care is concerningly lower in both markers of clinical care (e.g., use of effective medications) and cardiovascular disease control [2]. Such disparities are largely mediated by social determinants of health, in addition to biologic factors; therefore, multidimensional treatment approaches in chronic disease management among diverse populations are increasingly warranted [3].

The patient-centered medical home (PCMH) is widely promoted as a comprehensive health care delivery model that better organizes primary care at the population level [4], but its effect on racial-ethnic disparities remains unclear. PCMHs aim to improve access to a usual source of care through a primary care provider, which may level the playing field and reduce racial-ethnic disparities; yet, the model does not explicitly prioritize racial-ethnic disparities reduction in chronic disease care [5]. Smaller, regional studies have found that PCMH implementation may demonstrate racial-ethnic disparity reductions in preventive screenings [6] and even in patient outcomes related to diabetes management [7, 8]. Nonetheless, PCMHs have not been observed to narrow racial-ethnic disparity gaps in clinical care across diverse patient populations in larger, national studies of health systems [9–11].

It is not known whether persistent racial-ethnic disparities in health systems are similarly driven over time by the same set of multilevel factors. As PCMHs increase medical care accessibility, and new patients enter health systems to receive primary care, the sociodemographic characteristics and medical needs of primary care populations will change. Patients who newly engage with a health system after PCMH implementation may have greater clinical needs (may be “sicker”) than those who used it before, and subsequently, have worse health outcomes, such as poor hypertension and diabetes control. Conversely, PCMHs may increase access universally among patient groups and have no effect on reducing racial-ethnic disparities. Evaluations of PCMH outcomes must properly account for such patient-level factors to parse out differences that are related to patient clinical need (“just” or acceptable differences) versus differences related to true racial-ethnic disparity (“unjust” differences), as previously defined by the Institute of Medicine (IOM, now known as National Academy of Medicine)’s *Unequal Treatment* report.

While prior research points to healthcare access as a major influence on outcomes [12], studying PCMH outcomes in the Veterans Health Administration (VHA), the US’s largest integrated healthcare system, facilitates examination of additional contributing factors. Racial-ethnic disparities in clinical care continue to exist among primary care patients in the VHA [10]. While the US does not provide universal health coverage through a single-payer system, the VHA strives to lower financial barriers among US veterans and offers parity in care access once eligibility criteria are met [13]. In 2010, the VHA implemented its version of PCMHs across primary care practices nationally and found positive associations with several key outcomes: patient satisfaction, rates of hospitalization and emergency department use, quality of care, and staff burnout [14]. However, VHA’s implementation of PCMH had not been noted to narrow racial-ethnic gaps in hypertension and diabetes control among a national sample of veterans [10]. The contribution of non-clinical factors to overall disparities is unknown, along with whether contributors to disparities change over time. This follow-up study aims to identify and to compare underlying contributors to observed hypertension and diabetes control differences between minority, specifically Black and Hispanic, and White patients before (in 2009) and after (in 2014) VHA PCMH implementation.

## Materials and methods

This evaluation of racial-ethnic disparities in VHA received a Determination of Non-Research from the VA Greater Los Angeles Healthcare System Institutional Review Board. In a retrospective observational study, we examined hypertension and diabetes control among two independent probability samples of Black and Hispanic versus White primary care patients nationally in the VHA during fiscal years 2009 (October 1, 2008 to September 30, 2009; n_hypertension_ = 26,906; n_diabetes_ = 21,141) and 2014 (October 1, 2013 to September 30, 2014; n_hypertension_ = 83,809; n_diabetes_ = 38,887). We did not examine outcomes among smaller minority groups (i.e., American Indian/Alaska Native, Native Hawaiian/other Pacific Islander, Asian, multi-race) who were part of the original study [10]. Study patients had a VHA visit during the previous 13–24 months, as well as a VHA primary care visit during the 12 months prior and during the preceding month of sampling. Health records were sampled during both periods and reviewed by VHA’s quality monitoring program (External Peer Review Program) as administered by the Office of Performance Measurement within the VHA Office of Reporting, Analytics, Performance, Improvement & Deployment (Note larger sampling frame in 2014 than 2009). All data were fully anonymized before we accessed to them.

The main measures were two binary outcomes representing hypertension and diabetes control, as determined by trained VHA chart abstractors: blood pressure < 140/90 in patients with hypertension, and glycosylated hemoglobin (HbA1C) < = 9 in patients with diabetes. Both quality metrics are modeled after results reported in Healthcare Effectiveness Data and Information Set (HEDIS), which is one of US health care’s most widely used performance improvement tools.

The main independent variable was race-ethnicity (unknown for only 1% of the sample), obtained from multiple databases (i.e., VHA, Department of Defense). Individuals were categorized into the following groups: non-Hispanic White (White), non-Hispanic Black (Black), and Hispanic, which was classified based on reporting Hispanic ethnicity.

While we examined the same analytic sample of Black, Hispanic, and White patients as in our previous study [10], our covariates differed and were selected specifically to distinguish between racial-ethnic differences and disparities in quality of health care, as defined by the IOM [1]. Racial-ethnic differences consist of three distinct categories of effects from differences due to:

1. Clinical appropriateness and need and patient preferences
2. The operation of health care systems and the legal and regulatory climate, possibly related to socioeconomic status (SES)
3. Discrimination

The IOM considered only differences due to 2 and 3 be to be “unjust” differences, or racial-ethnic disparities. For 1, we examined age, sex, and a modified Seattle Index of Comorbidity [15]. This general measure of comorbidity examined seven chronic medical conditions (e.g., cancer, stroke, heart failure) and smoking status and was not included in the original study [10]. For 2, we approximated SES using the Area Deprivation Index (ADI), which uses 17 U.S. census poverty, education, housing, and employment indicators to characterize residential neighborhood [16, 17]. Individual level SES was not used in this study due to a high missing rate for that measure, but the ADI has been shown to approximate SES. Our models did not include variables for 3, which was presumed to account for racial-ethnic differences in disease control related to discrimination. We included fixed effects for Veterans Integrated Service Networks, which allowed us to account for regional variation among VHA facilities and hypertension and diabetes control rates.

We described rates of hypertension and diabetes control for each racial-ethnic patient group in each study year, as well as the change in control for each group from pre-to-post PCMH implementation. Furthermore, we analyzed patient characteristics for each racial-ethnic group over time, using t-tests for independent samples and χ2 tests of independence. We estimated linear probability regression models for racial-ethnic differences in hypertension and diabetes control for each study year, adjusting for age, sex, comorbidity, SES, and VHA region.

Anticipating statistically significant differences in hypertension and diabetes control between racial-ethnic minority and White patients in regression analyses, we first used the Blinder–Oaxaca decomposition method [18, 19] to parse out explained (attributable to study covariates) and unexplained components of the differences (possibly racial-ethnic discrimination). Previous health services researchers have used this method to study the contribution of underlying characteristics to racial-ethnic disparities in different health care quality measures [20–22]. This method further allowed us to decompose the percentages of the total racial-ethnic differences that are associated with differences in age, sex, comorbidity, SES, and unexplained (non-study) variables *at two separate time points* that spanned PCMH implementation.

Second, we used a technique for decomposing differences-in-differences (i.e., Smith-Welch decomposition) to understand whether the magnitudes of the explained and unexplained components of the racial-ethnic differences in outcomes changed *over time* with PCMH implementation. While not a new analytic approach in the social sciences fields [23, 24], this method of conducting a “difference-in-difference” decomposition, to our knowledge, has not been routinely used in health services research. As such, we used the Smith-Welch method to decompose the change in the magnitudes proportion of explained versus unexplained differences in hypertension and diabetes control over time and performed up to 1000 bootstrap replications, when able, to obtain standard errors.

All statistical tests were two-tailed, with statistical significance at an α of 0.0,5 and conducted all analyses in Stata 15.0 (College Station, TX).

## Results

Demographic and health characteristics differed among racial-ethnic groups in our two independent random samples of VA primary care patients with diabetes and/or hypertension before (2009) and after (2014) PCMH implementation. As described in the original study, racial-ethnic minority groups were younger, had higher or similar proportions of women, and had lower SES than White patients in both years [10]. For all racial-ethnic groups, there were a higher proportions of women and higher medical comorbidities after PCMH implementation than before. Also described in the original study, hypertension and diabetes control rates were lower for racial-ethnic minority versus White patient groups, and rates were statistically unchanged and stable for all racial-ethnic groups, in both years [10] (Table 1).

**Table 1:** Patient characteristics by racial-ethnic group before and after VHA patient-centered medical home implementation.

		Pre-PCMH (FY2009)					Post-PCMH (FY2014)				
**Hypertension**		White	Black		Hispanic		White	Black		Hispanic	
		(n = 21,441)	(n = 4,566)		(n = 899)		(n = 64,270)	(n = 15,952)		(n = 3,587)	
Characteristics											
Sex	*Female*	2.70%	6.30%	***	2.00%		11.20%	24.70%	***	10.70%	
Age	*18–44*	3.00%	6.50%	***	7.50%	***	3.40%	7.30%	***	6.90%	***
*45–64*		44.50%	65.00%	***	58.10%	***	38.50%	60.40%	***	48.90%	***
*65+*		52.40%	28.50%	***	34.50%	***	58.10%	32.40%	***	44.20%	***
Comorbidity† (mean/SD)		2.69 (2.37)	2.77 (2.41)		2.53 (2.29)	*	3.17 (2.58)	3.08 (2.55)	***	2.81 (2.29)	***
Socioeconomic status‡ (mean/SD)		102.46 (13.68)	107.08 (11.79)	***	104.38 (12.4)	***	102.87 (13.04)	106.09 (12.16)	***	103.22 (14.82)	
Chronic Disease Control											
Blood pressure <140/90		79.00%	73.40%	***	74.80%	**	77.70%	71.70%	***	75.60%	**
**Diabetes**		White	Black		Hispanic		White	Black		Hispanic	
		(n = 16,256)	(n = 3,848)		(n = 1,037)		(n = 29,406)	(n = 7,384)		(n = 2,097)	
Characteristics											
Sex	*Female*	2.50%	4.70%	***	1.50%	***	7.00%	15.70%	***	7.80%	***
Age	*18–44*	2.40%	5.00%	***	4.90%	***	2.40%	4.70%	***	6.30%	***
*45–64*		59.70%	72.70%	***	70.30%	***	42.40%	62.30%	***	52.70%	***
*65+*		38.00%	22.20%	***	24.80%	***	55.20%	33.00%	***	41.10%	***
Comorbidity† (mean/SD)		3.89 (2.21)	3.80 (2.16)	*	3.43 (1.97)	*	4.13 (2.38)	4.11 (2.38)	***	3.56 (2.08)	***
Socioeconomic status‡ (mean/SD)		103.42 (12.21)	106.67 (11.87)	***	104.26 (13.39)	***	103.22 (12.3)	105.99 (12.34)	***	103.55 (14.26)	***
Chronic Disease Control											
Hemoglobin A1c <9		86.50%	79.30%	***	78.50%	***	82.40%	76.10%	***	76.50%	***

Differences between minority and White groups were tested for significance using χ^2^ tests of independence (for patient outcomes hypertension and diabetes control; age; sex) or t-tests for independent samples (comorbidity, socioeconomic status). Area deprivation index higher values indicate greater neighborhood deprivation (i.e., lower socioeconomic status). SD = standard deviation.

*p<0.05

**p<0.01

***p<0.001

†measured by the Modified Seattle Comorbidity Index

‡ measured by the Area Deprivation Index.

In fully adjusted models, compared to White patients, in 2009, hypertension and diabetes control remained significantly lower for Black (-5.7%[0.8%] and -4.7%[0.7%], respectively; p’s<0.001) and Hispanic (-3.8%[1.5%] and -5.7%[1.2%], respectively; p’s<0.001) patients. In 2014, compared to White patients, hypertension and diabetes control remained significantly lower for Black (-6.2%[0.4%] and -3.1%[0.6%], respectively; p’s<0.001) and Hispanic (-1.4%[0.8%] and -4.0%[1.0%], respectively; p’s<0.001) patients. While our covariates differed from the original study, all were significant predictors of disease control in study models. Our current models similarly documented persistent gaps in hypertension and diabetes control between racial-ethnic minority and White patient groups, so we proceeded to parse out explained (by covariates) and unexplained (e.g., discrimination) contributors to racial-ethnic differences.

In decomposition analyses, we found that most racial-ethnic differences (55.7–92.3%; p’s<0.05) were *not* attributed to age, sex, comorbidity, and SES (explained). The proportion of explained relative to overall difference between minority versus White patient groups was small for hypertension. Only 10.4% (age [6.1%], SES [4.6%]) of the Black-White and 6.0% (age [4.6%], comorbidity [1.4%]) of the Hispanic-White difference in hypertension control was explained in 2009. Only 7.6% (age [7.3%], sex [-2.1%], comorbidity [0.4%], SES [2.1%]) of the Black-White and 17.3% (age [11.8%], comorbidity [4.9%], SES [0.5%]) of the Hispanic-White difference was explained in 2014 (p’s<0.05). The proportion of explained relative to overall difference was slightly larger for diabetes than hypertension. 32.5% (age [30.2%], SES [2.3%]) of the Black-White and 19.9% (age [19.3%], SES [0.6%]) of the Hispanic-White difference in diabetes control was explained in 2009. 45.7% (age [43.9%], comorbidity [-0.5%], SES [2.2%]) of the Black-White and 29.9% (age [32.2%], comorbidity [-2.6%], SES [0.2%]) of the Hispanic-White difference was explained in 2014 (p’s<0.05) (Table 2).

**Table 2:** Decomposing racial-ethnic disparities in hypertension and diabetes control before and after VHA patient-centered medical home implementation.

			Black-White				Hispanic-White			
			Pre-PCMH (FY2009)		Post-PCMH (FY2014)		Pre-PCMH (FY2009)		Post-PCMH (FY2014)	
**Hypertension**				p-value		p-value		p-value		p-value
Difference (SE)			0.056 (0.01)	<0.001***	0.060 (0.00)	<0.001***	0.043 (0.01)	<0.001***	0.021 (0.01)	<0.001***
	Explained		0.007 (0.00)	<0.001***	0.005 (0.00)	<0.001***	0.004 (0.00)	<0.001***	0.004 (0.00)	<0.001***
		Age	0.003 (0.00)	0.02*	0.004 (0.00)	<0.001***	0.003 (0.00)	0.03*	0.003 (0.00)	<0.001***
		Sex	0.001 (0.00)	0.22	-0.001 (0.00)	0.04*	0.000 (0.00)	0.38	0.000 (0.00)	0.60
		Comorbidity	0.000 (0.00)	0.16	0.000 (0.00)	<0.001***	0.001 (0.00)	0.09	0.001 (0.00)	<0.001***
		SES	0.003 (0.00)	<0.001***	0.001 (0.00)	<0.001***	0.001 (0.00)	0.10	0.000 (0.00)	0.23
	Unexplained		0.050 (0.001)	<0.001***	0.056 (0.00)	<0.001***	0.039 (0.01)	0.01**	0.018 (0.01)	0.02*
**Diabetes**										
Difference (SE)			0.072 (0.01)	<0.001***	0.064 (0.001)	<0.001***	0.080 (0.01)	<0.001***	0.059 (0.01)	<0.001***
	Explained		0.023 (0.00)	<0.001***	0.028 (0.000)	<0.001***	0.016 (0.00)	<0.001***	0.018 (0.00)	<0.001***
		Age	0.022 (0.00)	<0.001***	0.028 (0.000)	<0.001***	0.015 (0.00)	<0.001***	0.019 (0.00)	<0.001***
		Sex	0.000 (0.00)	0.96	-0.001 (0.000)	0.21	0.000 (0.00)	0.32	0.000 (0.00)	0.45
		Comorbidity	0.000 (0.00)	0.32	0.000 (0.000)	0.02*	0.000 (0.00)	0.70	-0.002 (0.00)	<0.001***
		SES	0.002 (0.00)	0.01**	0.001 (0.000)	<0.001***	0.000 (0.00)	0.11	0.000 (0.00)	0.33
	Unexplained		0.048 (0.01)	<0.001***	0.035 (0.001)	<0.001***	0.064 (0.01)	<0.001***	0.041 (0.01)	<0.001***

The Blinder-Oaxaca decomposition method parsed out explained (by variables in the regression models) and unexplained (e.g., discrimination) contributors to racial-ethnic differences in patient outcomes, as shown by the coefficients and standard errors (SE) in this Table. In the Results text, we depict these findings as proportions relative to the overall difference (i.e., explained/difference). Note that the proportion of explained and unexplained contributors did not significantly change from FY2009 to FY2014.

*p<0.05

**p<0.01

***p<0.001.

In our “difference-in-differences (DID)” decomposition analyses, we did not find significant changes over time in the proportion of contributing factors to racial-ethnic disparities in hypertension outcomes.

## Discussion

Racial-ethnic disparities in hypertension and diabetes control among veterans using VHA care persisted despite PCMH implementation in our current study, similar to our original findings [10]. When we further decomposed racial-ethnic differences in chronic disease control over time, we found no significant changes in the contribution of explained (i.e., age, sex, comorbidity, SES) versus unexplained factors (e.g., discrimination), despite PCMH implementation aiming to improve care access in the VHA. While many explanations for persistent racial-ethnic disparities may exist [25], we did not find that it was due to an influx of “sick” or “socioeconomically vulnerable” patients into the VHA. Instead, all-around high patient volume (i.e., increase from 5.1 million to 5.8 million VHA patients) [10] may have outpaced the low supply of primary care staff and resulted in less effective PCMH implementation [14]. Therefore, these competing demands may make it difficult for PCMH models to focus on racial-ethnic disparities reduction in chronic disease care. To start, PCMH models may consider explicitly incentivizing racial-ethnic disparities reduction in chronic disease care [5, 26], such as increasing mental health services for minority patients [27], and investigating which individual PCMH components is linked with improved disparities [28]. Regardless, more research is needed to understand the relationship between PCMH models, and their variable implementations, and care quality over time among different patient groups, given the racial-ethnic inequity that remains post-implementation.

Overall, we found poor progress in narrowing racial-ethnic inequities among primary care patients with chronic diseases in our study and that unexplained factors accounted for most of the minority-White differences observed. Unexplained differences, which are not explained by our study variables (i.e., age, sex, comorbidity, SES), may possibly be due to ongoing differential healthcare and community experiences (e.g., discrimination). Literature on racial-ethnic inequity and PCMH models from the US and other countries suggest that disadvantaged patients (e.g., poor, immigrant) were less likely to reap the benefits of PCMH implementation [29–31]. In fact, VHA facilities with higher racial-ethnic minority composition, where most Black and Hispanic patient groups receive care, have also been found to demonstrate lower PCMH implementation progress than facilities with lower minority composition [32]. Racial-ethnic minority veterans are consequently less likely to benefit from PCMH care enhancements. Discrimination is unlikely to be isolated to one specific component of healthcare delivery (e.g., implicit racial-ethnic bias from clinicians, medication non-adherence due to historical mistrust of healthcare systems by minorities). Efforts to eliminate disparities could comprehensively target improvements in outcomes among Black and Hispanic patients [5, 26] and begin by prioritizing quality improvement efforts in facilities with high minority patient composition.

While our study boasts two large national samples at two timepoints within an integrated health delivery system, it is affected by a few limitations. First is our inability to discount effects of competing policies, such as the broader US enactment of the Affordable Care Act to increase health insurance access, during this time period. To our knowledge, however, PCMH implementation was the only major VHA initiative that redesigned primary care practices with documented positive effects on hypertension and diabetes controls among Veterans [14]. Second, we are unable to discern exactly what accounts for unobserved factors driving racial-ethnic disparities in chronic disease control and can only posit that differential experiences (e.g., discrimination) both within and outside of healthcare settings is involved. Researchers in economics [18, 19] and health services research [20–22] have drawn similar conclusions regarding discrimination using decomposition analyses in the past. More research is needed to rule-out confounding patient-level factors, such as patient preference [33], and to characterize components of differential experiences that may affect chronic disease control (e.g., greater therapeutic inertia and communication barriers by providers for racial-ethnic minorities, associated disparities in trust in provider affecting medication adherence) [34–36]. To address this, future research should consider merging other data sources (e.g., pharmacy) or collecting new ones (e.g., patient-reported outcomes) in decomposition analyses. Third, study generalizability may be limited to US veterans who receive care within the VHA, who are often older, predominantly male, with higher disease burden population, and has access to healthcare [37].

## Conclusions

Our study aims to move the health inequity discussion beyond merely describing the presence of racial-ethnic disparities in chronic disease control within PCMHs to an understanding of the underlying pathways leading to such disparities and any explanatory changes over time. Despite aiming to improve care access through VHA PCMH implementation, racial-ethnic differences in hypertension and diabetes control between Black and Hispanic versus White patients persisted. Differences remained largely unexplained by age, sex, comorbidity, and SES and may possibly be due to differential healthcare and community experiences (e.g., discrimination). Targeted outreach to vulnerable populations (e.g., tailored interventions for aging Black Veterans for hypertension or Hispanic Veterans with multiple comorbidities for diabetes), and at facilities with high minority composition, may offer greatest improvements to hypertension and diabetes control equity. When employed in large health program evaluations, “difference-in-difference” decomposition analyses can examine whether racial-ethnic health disparities—an important element of care quality—improve as a result of program implementation. Understanding underlying pathways that lead to health disparities will better inform policy and clinical interventions to improve PCMH care delivery to minority patients in the VHA and other healthcare delivery systems.
